# A new form of NaMnAsO_4_

**DOI:** 10.1107/S2056989019008065

**Published:** 2019-06-07

**Authors:** Matthias Weil, Théo Veyer

**Affiliations:** a Institute for Chemical Technologies and Analytics, Division of Structural Chemistry, TU Wien, Getreidemarkt 9/164-SC, A-1060 Vienna, Austria; b IUT Bordeaux 1, 15 Rue Naudet, 33175 Gradignan, France

**Keywords:** crystal structure, dimorphism, structure comparison, hydro­thermal synthesis

## Abstract

A new form of NaMnAsO_4_, sodium manganese(II) orthoarsenate, has been obtained under hydro­thermal conditions, and is referred to as the β-polymorph. In contrast to the previously reported ortho­rhom­bic α-polymorph that crystallizes in the olivine-type of structure and has one manganese(II) cation in a distorted octa­hedral coordination, the current β-polymorph contains two manganese(II) cations in [5]-coordination, inter­mediate between a square-pyramid and a trigonal bipyramid. In the crystal structure of β-NaMnAsO_4_, four [MnO_5_] polyhedra are linked through vertex- and edge-sharing into finite {Mn_4_O_16_} units strung into rows parallel to [100]. These units are linked through two distinct orthoarsenate groups into a framework structure with channels propagating parallel to the manganese oxide rows. Both unique sodium cations are situated inside the channels and exhibit coordination numbers of six and seven. β-NaMnAsO_4_ is isotypic with one form of NaCoPO_4_ and with NaCuAsO_4_.

## Abstract 2

The new form of NaMnAsO_4_ (denoted as β) crystallizes in the monoclinic crystal system and is isotypic with one form of NaCoPO_4_ and with NaCuAsO_4_.

## Chemical context   

Magnussonite is a rare manganese(II) arsenite mineral and has been described with an ideal formula of Mn^II^_10_As^III^_6_O_18_(OH,Cl)_2_ (Moore & Araki, 1979 ▸). In a recent project on hydro­thermal crystal growth of phases in the system Mn^II^/As^III^/O (Priestner *et al.*, 2018*a* ▸) and a precise structure refinement of magnussonite, it could be shown that the obtained synthetic material has a composition of Mn^II^_3_As^III^_2_O_6_·1/3H_2_O whereas naturally occurring material (type locality Långban, Sweden) is better described as Mn^II^_3_As^III^_2_O_6_(Cu^II^(OH,Cl)_2_)_*x*_ (Priestner *et al.*, 2018*b* ▸). Building on that knowledge, a subsequent project was started to incorporate divalent transition-metal cations under hydro­thermal conditions into synthetic magnussonite for obtaining similar compositions to those in the natural material. In one of the batches, containing manganese(II) acetate, sodium hydroxide, nickel chloride and arsenic(III) oxide as the arsenic source, we observed a partial oxidation of arsenic to yield monoclinic NaMnAsO_4_ as a by-product with arsenic in an oxidation state of +V. NaMnAsO_4_ was reported previously, as obtained from a high-temperature synthesis in a molten salt medium (Ulutagay-Kartin *et al.*, 2002 ▸). This form crystallizes in the ortho­rhom­bic system with space-group type *Pnma* and adopts an olivine-type of structure.

In the following, we refer to the previously reported ortho­rhom­bic polymorph (Ulutagay-Kartin *et al.*, 2002 ▸) as the α-form, and the new monoclinic polymorph as the β-form of NaMnAsO_4_.

## Structural commentary   

**Figure 1:**
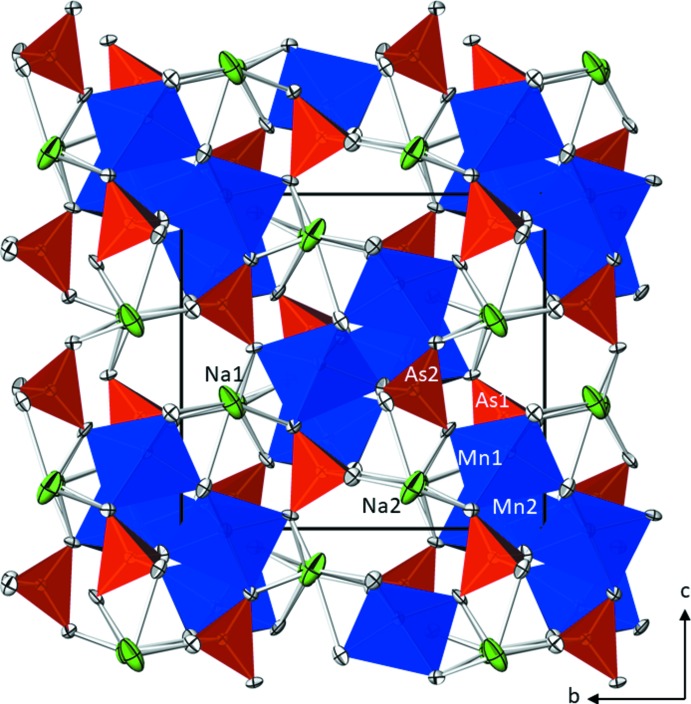
The crystal structure of β-NaMnAsO_4_ in a projection along [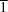
00]. [MnO_5_] polyhedra are shown in blue, [AsO_4_] tetra­hedra in red, Na^I^ cations in green and O atoms in shaded grey. Displacement ellipsoids are drawn at the 90% probability level.

β-NaMnAsO_4_ crystallizes isotypically with one of the three modifications of the phosphate NaCoPO_4_ (Feng *et al.*, 1997 ▸) and with the copper analogue NaCuAsO_4_ (Ulutagay-Kartin *et al.*, 2003 ▸). The asymmetric unit of β-NaMnAsO_4_ comprises of two formula units, and the principal building units are two manganese(II) cations in [5]-coordination, two orthoarsenate anions AsO_4_^3–^, and two sodium cations in a six- and sevenfold coordination by oxygen (Fig. 1 ▸).

The *τ*_5_ descriptor (Addison *et al.*, 1984 ▸) was calculated as 0.59 for the polyhedron around Mn1 and 0.54 for that around Mn2, meaning that the shapes of the polyhedra are inter­mediate between a square pyramid (*τ*_5_ = 0) and a trigonal bipyramid (*τ*_5_ = 1). The Mn—O bond lengths range from 2.061 (3)–2.205 (3) Å whereby those involving Mn1 scatter in a greater range than those involving Mn2 (Table 1 ▸). Two [Mn2O_5_] polyhedra are fused into a centrosymmetric dimer by sharing an edge. To each side of the dimer two [Mn1O_5_] polyhedra are attached by sharing a common vertex, thus establishing a finite {Mn_4_O_16_} unit. In the crystal structure of the α-polymorph (Ulutagay-Kartin *et al.*, 2002 ▸), the unique Mn^II^ site has a distorted octa­hedral environment with bond lengths ranging from 2.121 (2)–2.339 (2) Å. Here the [MnO_6_] units are connected through sharing four of their vertices into perovskite-type sheets. The isolated {Mn_4_O_16_} units in the β-polymorph are strung into rows parallel to [100] and are connected into a three-dimensional framework structure by AsO_4_^3–^ tetra­hedra sharing common vertices. The As—O bond lengths (Table 1 ▸) are characteristic for isolated orthoarsenate groups, and their mean values of 1.692 Å (As1) and 1.676 Å (As2) conform with literature data (1.687 Å; Gagné & Hawthorne, 2018 ▸). This framework delimits channels parallel to [100] in which the two sodium cations are situated. They are surrounded by six (Na1) and seven (Na2) oxygen atoms, each displaying a distorted coordination polyhedron. Relevant Na—O distances are collated in Table 1 ▸. The results of bond-valence-sum calculations (Brown, 2002 ▸; Brese & O’Keeffe, 1991 ▸) are consistent with the expected oxidation states of +I for Na, +II for Mn, +V for As, and −II for O (values in valence units): Na1 = 1.03, Na2 = 0.95, Mn1 = 1.94, Mn2 = 1.98, As1 = 4.90, As2 = 5.12, O atoms = 1.83–2.16.

The previously reported α-form of NaMnAsO_4_ has a calculated X-ray density *D_x_* = 4.03 g cm^−3^ and thus is denser than the current β-form (3.95 g cm^−3^). Based on the rule of thumb that the denser polymorph is (in the majority of cases) the stable form, these values point to α-NaMnAsO_4_ as the thermodynamically stable polymorph. This assumption is supported by the preparation conditions of the different polymorphs. The α-polymorph was obtained under high-temperature conditions (Ulutagay-Kartin *et al.*, 2002 ▸) whereas the β-polymorph crystallized under much milder temperature conditions. As a result of the scarcity of β-NaMnAsO_4_ material, a detailed investigation of the thermal behaviour was not conducted. However, a possible β → α phase transition would be of the reconstructive type because the building units in the two structures exhibit a completely different arrangement.

For a qu­anti­tative structural comparison of β-NaMnAsO_4_ with the isotypic sodium copper(II) arsenate analogue, NaCuAsO_4_ (Ulutagay-Kartin *et al.*, 2003 ▸), the program *compstru* (de la Flor *et al.*, 2016 ▸) available at the Bilbao Crystallographic Server (Aroyo *et al.*, 2006 ▸) was used. The comparison revealed a degree of lattice distortion of 0.0170, the maximum distance between the atomic positions of paired atoms of 0.1834 Å for pair Na1, the arithmetic mean of all distances of 0.1150 Å, and the measure of similarity of 0.050. All these values show a high similarity between the two crystal structures. This is supported by the similar *τ*_5_ values of 0.57 and 0.47 for the two copper(II) cations in NaCuAsO_4_.

## Synthesis and crystallization   

A stoichiometric mixture of Mn(CH_3_COO)_2_·4H_2_O, NaOH, As_2_O_3_ and NiCl_2_ in the ratio 1:6:1:1/6 was loaded in a Teflon container that was filled with 3 ml of water to two-thirds of its volume. Then the container was sealed with a Teflon lid and placed in a steel autoclave that was heated at 483 K for five days. After cooling to room temperature, the solid material was filtered off, washed with mother liquor, water and ethanol and air-dried. The main phase identified by single crystal and powder X-ray diffraction was synthetic magnussonite, Mn_3_As_2_O_6_·1/3H_2_O (Priestner *et al.*, 2018*b* ▸). Synthetic sarkinite, a basic manganese(II) arsenate(V) with formula Mn_2_AsO_4_(OH) (Stock *et al.*, 2002 ▸), and the title compound were also present as minor by-products, with β-NaMnAsO_4_ typically appearing in the form of needles.

## Refinement   

Crystal data, data collection and structure refinement details are summarized in Table 2 ▸. Coordinates of isotypic NaCuAsO_4_ (Ulutagay-Kartin *et al.*, 2003 ▸) were standardized using the program *STRUCTURE-TIDY* (Gelato & Parthé, 1987 ▸) and then used as starting parameters for refinement. Free refinement of the site occupation factors for the two Mn sites resulted in a value of 1.000 (3) in each case, thus revealing no incorporation of Ni at these sites.

## Supplementary Material

Crystal structure: contains datablock(s) I, global. DOI: 10.1107/S2056989019008065/vn2148sup1.cif

Structure factors: contains datablock(s) I. DOI: 10.1107/S2056989019008065/vn2148Isup2.hkl

CCDC reference: 1921163

Additional supporting information:  crystallographic information; 3D view; checkCIF report

## Figures and Tables

**Table 1:** Selected bond lengths (Å)

Na1—O6^i^	2.368 (3)	Mn1—O7	2.162 (3)
Na1—O1^ii^	2.407 (3)	Mn1—O3	2.205 (3)
Na1—O2^iii^	2.411 (3)	Mn2—O2^v^	2.095 (3)
Na1—O5^ii^	2.496 (3)	Mn2—O4^vii^	2.124 (3)
Na1—O3^i^	2.526 (4)	Mn2—O1	2.139 (3)
Na1—O7^iv^	2.547 (3)	Mn2—O8^v^	2.150 (3)
Na2—O3	2.376 (3)	Mn2—O4^v^	2.155 (3)
Na2—O2^v^	2.409 (4)	As1—O2	1.683 (3)
Na2—O7^i^	2.539 (3)	As1—O5^vii^	1.689 (3)
Na2—O5	2.540 (3)	As1—O1	1.694 (3)
Na2—O1	2.580 (4)	As1—O7	1.703 (3)
Na2—O6	2.712 (4)	As2—O6	1.647 (3)
Na2—O8	2.829 (4)	As2—O3^viii^	1.676 (3)
Mn1—O6	2.061 (3)	As2—O4^ix^	1.684 (3)
Mn1—O5^vi^	2.144 (3)	As2—O8	1.696 (3)
Mn1—O8^vi^	2.144 (3)		

Symmetry codes: (i) 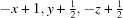; (ii) 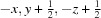; (iii) 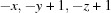; (iv) 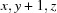; (v) 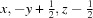; (vi) 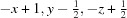; (vii) 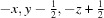; (viii) 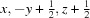; (ix) 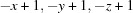.

**Table 2:** Experimental details

Crystal data	
Chemical formula	NaMnAsO_4_
*M*_r_	216.85
Crystal system, space group	Monoclinic, *P*2_1_/*c*
Temperature (K)	296
*a*, *b*, *c* (Å)	6.0917 (6), 11.4072 (10), 10.5008 (9)
β (°)	91.517 (3)
*V* (Å^3^)	729.44 (11)
*Z*	8
Radiation type	Mo *K*α
μ (mm^−1^)	12.60
Crystal size (mm)	0.12 × 0.02 × 0.01

Data collection	
Diffractometer	Bruker APEXII CCD
Absorption correction	Multi-scan (*SADABS*; Krause *et al.*, 2015 ▸)
*T*_min_, *T*_max_	0.476, 0.746
No. of measured, independent and observed [*I* > 2σ(*I*)] reflections	10811, 2336, 1682
*R*_int_	0.075
(sin θ/λ)_max_ (Å^−1^)	0.726

Refinement	
*R*[*F* ^2^ > 2σ(*F* ^2^)], *wR*(*F* ^2^), *S*	0.035, 0.059, 1.00
No. of reflections	2336
No. of parameters	128
Δρ_max_, Δρ_min_ (e Å^−3^)	0.93, −0.94

Computer programs: *APEX3* and *SAINT* (Bruker, 2015 ▸), *SHELXL2017* (Sheldrick, 2015 ▸), *ATOMS* (Dowty, 2006 ▸) and *publCIF* (Westrip, 2010 ▸).

## supplementary crystallographic information

### Crystal data

**Table:**

NaMnAsO_4_	*F*(000) = 808
*M**_r_* = 216.85	*D*_x_ = 3.949 Mg m^−^^3^
Monoclinic, *P*2_1_/*c*	Mo *K*α radiation, λ = 0.71073 Å
*a* = 6.0917 (6) Å	Cell parameters from 1472 reflections
*b* = 11.4072 (10) Å	θ = 2.6–29.1°
*c* = 10.5008 (9) Å	µ = 12.60 mm^−^^1^
β = 91.517 (3)°	*T* = 296 K
*V* = 729.44 (11) Å^3^	Needle, colourless
*Z* = 8	0.12 × 0.02 × 0.01 mm

### Data collection

**Table:**

Bruker APEXII CCD diffractometer	1682 reflections with *I* > 2σ(*I*)
ω– and φ–scans	*R*_int_ = 0.075
Absorption correction: multi-scan (*SADABS*; Krause *et al.*, 2015)	θ_max_ = 31.1°, θ_min_ = 3.4°
*T*_min_ = 0.476, *T*_max_ = 0.746	*h* = −8→8
10811 measured reflections	*k* = −16→16
2336 independent reflections	*l* = −15→15

### Refinement

**Table:**

Refinement on *F*^2^	0 restraints
Least-squares matrix: full	*w* = 1/[σ^2^(*F*_o_^2^) + (0.0138*P*)^2^] where *P* = (*F*_o_^2^ + 2*F*_c_^2^)/3
*R*[*F*^2^ > 2σ(*F*^2^)] = 0.035	(Δ/σ)_max_ < 0.001
*wR*(*F*^2^) = 0.059	Δρ_max_ = 0.93 e Å^−^^3^
*S* = 1.00	Δρ_min_ = −0.94 e Å^−^^3^
2336 reflections	Extinction correction: SHELXL2017 (Sheldrick, 2015), Fc^*^=kFc[1+0.001xFc^2^λ^3^/sin(2θ)]^-1/4^
128 parameters	Extinction coefficient: 0.00083 (19)

### Special details

**Table:**

Geometry. All esds (except the esd in the dihedral angle between two l.s. planes) are estimated using the full covariance matrix. The cell esds are taken into account individually in the estimation of esds in distances, angles and torsion angles; correlations between esds in cell parameters are only used when they are defined by crystal symmetry. An approximate (isotropic) treatment of cell esds is used for estimating esds involving l.s. planes.

### Fractional atomic coordinates and isotropic or equivalent isotropic displacement parameters (Å^2^)

**Table:**

	*x*	*y*	*z*	*U*_iso_*/*U*_eq_	
As1	0.12080 (7)	0.11305 (3)	0.33114 (4)	0.00717 (10)	
As2	0.62792 (7)	0.35882 (3)	0.41290 (4)	0.00749 (10)	
Mn1	0.61290 (11)	0.09632 (5)	0.23084 (6)	0.00943 (14)	
Mn2	0.14942 (11)	0.12113 (5)	0.00555 (6)	0.00920 (14)	
Na1	0.1190 (3)	0.84466 (15)	0.36519 (18)	0.0193 (4)	
Na2	0.3740 (3)	0.36374 (17)	0.1217 (2)	0.0294 (5)	
O1	0.1342 (5)	0.1909 (2)	0.1942 (3)	0.0135 (7)	
O2	0.1143 (5)	0.2010 (2)	0.4596 (3)	0.0117 (6)	
O3	0.5901 (5)	0.2026 (3)	0.0556 (3)	0.0148 (7)	
O4	0.1471 (5)	0.5556 (2)	0.5782 (3)	0.0111 (6)	
O5	0.1034 (5)	0.5259 (2)	0.1712 (3)	0.0123 (6)	
O6	0.6572 (5)	0.2657 (2)	0.2946 (3)	0.0145 (7)	
O7	0.3513 (5)	0.0291 (2)	0.3453 (3)	0.0117 (6)	
O8	0.3980 (5)	0.4378 (2)	0.3788 (3)	0.0117 (6)	

### Atomic displacement parameters (Å^2^)

**Table:**

	*U*^11^	*U*^22^	*U*^33^	*U*^12^	*U*^13^	*U*^23^
As1	0.0078 (2)	0.0068 (2)	0.0069 (2)	−0.00017 (16)	−0.00021 (16)	−0.00042 (16)
As2	0.0079 (2)	0.0072 (2)	0.0073 (2)	−0.00031 (16)	−0.00027 (16)	0.00011 (16)
Mn1	0.0117 (3)	0.0073 (3)	0.0093 (3)	−0.0010 (2)	−0.0009 (2)	−0.0003 (2)
Mn2	0.0104 (3)	0.0079 (3)	0.0094 (3)	0.0007 (2)	0.0008 (2)	0.0000 (2)
Na1	0.0179 (10)	0.0133 (9)	0.0271 (11)	0.0000 (8)	0.0083 (8)	−0.0007 (8)
Na2	0.0195 (10)	0.0250 (11)	0.0432 (13)	0.0032 (8)	−0.0086 (9)	−0.0179 (9)
O1	0.0214 (17)	0.0099 (14)	0.0094 (17)	0.0012 (13)	0.0032 (13)	0.0014 (11)
O2	0.0176 (17)	0.0121 (14)	0.0055 (15)	0.0001 (13)	0.0038 (12)	−0.0022 (11)
O3	0.0245 (18)	0.0115 (15)	0.0082 (16)	0.0030 (13)	0.0001 (13)	0.0001 (12)
O4	0.0068 (15)	0.0102 (14)	0.0161 (17)	−0.0040 (12)	−0.0019 (12)	−0.0019 (12)
O5	0.0088 (16)	0.0123 (15)	0.0157 (17)	0.0025 (12)	−0.0002 (12)	−0.0013 (12)
O6	0.0217 (18)	0.0104 (15)	0.0115 (17)	−0.0022 (12)	0.0028 (13)	−0.0064 (12)
O7	0.0083 (15)	0.0127 (15)	0.0141 (16)	0.0028 (12)	0.0005 (12)	0.0015 (12)
O8	0.0075 (15)	0.0114 (15)	0.0161 (17)	0.0024 (12)	0.0005 (12)	0.0039 (12)

### Geometric parameters (Å, º)

**Table:**

Na1—O6^i^	2.368 (3)	Mn1—O7	2.162 (3)
Na1—O1^ii^	2.407 (3)	Mn1—O3	2.205 (3)
Na1—O2^iii^	2.411 (3)	Mn2—O2^v^	2.095 (3)
Na1—O5^ii^	2.496 (3)	Mn2—O4^vii^	2.124 (3)
Na1—O3^i^	2.526 (4)	Mn2—O1	2.139 (3)
Na1—O7^iv^	2.547 (3)	Mn2—O8^v^	2.150 (3)
Na2—O3	2.376 (3)	Mn2—O4^v^	2.155 (3)
Na2—O2^v^	2.409 (4)	As1—O2	1.683 (3)
Na2—O7^i^	2.539 (3)	As1—O5^vii^	1.689 (3)
Na2—O5	2.540 (3)	As1—O1	1.694 (3)
Na2—O1	2.580 (4)	As1—O7	1.703 (3)
Na2—O6	2.712 (4)	As2—O6	1.647 (3)
Na2—O8	2.829 (4)	As2—O3^viii^	1.676 (3)
Mn1—O6	2.061 (3)	As2—O4^ix^	1.684 (3)
Mn1—O5^vi^	2.144 (3)	As2—O8	1.696 (3)
Mn1—O8^vi^	2.144 (3)		
			
O2—As1—O5^vii^	109.07 (14)	O3—Na2—O6	61.90 (10)
O2—As1—O1	111.74 (14)	O2^v^—Na2—O6	137.80 (12)
O5^vii^—As1—O1	110.68 (15)	O7^i^—Na2—O6	79.02 (11)
O2—As1—O7	107.60 (15)	O5—Na2—O6	124.60 (12)
O5^vii^—As1—O7	109.56 (14)	O1—Na2—O6	80.96 (11)
O1—As1—O7	108.11 (14)	O3—Na2—O8	119.67 (12)
O6—As2—O3^viii^	115.17 (14)	O2^v^—Na2—O8	141.48 (13)
O6—As2—O4^ix^	108.13 (15)	O7^i^—Na2—O8	68.33 (10)
O3^viii^—As2—O4^ix^	108.90 (15)	O5—Na2—O8	66.72 (10)
O6—As2—O8	106.78 (15)	O1—Na2—O8	87.83 (11)
O3^viii^—As2—O8	106.17 (15)	O6—Na2—O8	57.89 (9)
O4^ix^—As2—O8	111.73 (14)	As1—O1—Mn2	126.51 (15)
O6—Mn1—O5^vi^	95.67 (12)	As1—O1—Na1^vii^	123.87 (15)
O6—Mn1—O8^vi^	165.34 (12)	Mn2—O1—Na1^vii^	94.27 (12)
O5^vi^—Mn1—O8^vi^	87.40 (11)	As1—O1—Na2	133.73 (16)
O6—Mn1—O7	104.14 (12)	Mn2—O1—Na2	88.43 (11)
O5^vi^—Mn1—O7	101.37 (11)	Na1^vii^—O1—Na2	74.38 (10)
O8^vi^—Mn1—O7	89.23 (11)	As1—O2—Mn2^viii^	139.26 (16)
O6—Mn1—O3	76.13 (11)	As1—O2—Na2^viii^	110.78 (15)
O5^vi^—Mn1—O3	129.69 (11)	Mn2^viii^—O2—Na2^viii^	94.20 (12)
O8^vi^—Mn1—O3	90.86 (11)	As1—O2—Na1^iii^	120.64 (15)
O7—Mn1—O3	128.90 (12)	Mn2^viii^—O2—Na1^iii^	95.30 (11)
O2^v^—Mn2—O4^vii^	99.50 (12)	Na2^viii^—O2—Na1^iii^	77.51 (11)
O2^v^—Mn2—O1	81.14 (11)	As2^v^—O3—Mn1	120.63 (15)
O4^vii^—Mn2—O1	117.21 (12)	As2^v^—O3—Na2	132.47 (17)
O2^v^—Mn2—O8^v^	103.27 (11)	Mn1—O3—Na2	101.81 (12)
O4^vii^—Mn2—O8^v^	103.81 (11)	As2^v^—O3—Na1^vi^	116.79 (15)
O1—Mn2—O8^v^	137.57 (12)	Mn1—O3—Na1^vi^	92.86 (12)
O2^v^—Mn2—O4^v^	170.23 (11)	Na2—O3—Na1^vi^	78.27 (11)
O4^vii^—Mn2—O4^v^	78.69 (12)	As2^ix^—O4—Mn2^ii^	120.06 (15)
O1—Mn2—O4^v^	91.11 (11)	As2^ix^—O4—Mn2^viii^	123.30 (15)
O8^v^—Mn2—O4^v^	86.45 (11)	Mn2^ii^—O4—Mn2^viii^	101.31 (12)
O6^i^—Na1—O1^ii^	85.20 (12)	As1^ii^—O5—Mn1^i^	115.32 (14)
O6^i^—Na1—O2^iii^	145.14 (12)	As1^ii^—O5—Na1^vii^	92.86 (13)
O1^ii^—Na1—O2^iii^	69.73 (11)	Mn1^i^—O5—Na1^vii^	144.25 (14)
O6^i^—Na1—O5^ii^	121.79 (12)	As1^ii^—O5—Na2	162.63 (17)
O1^ii^—Na1—O5^ii^	102.84 (12)	Mn1^i^—O5—Na2	81.48 (10)
O2^iii^—Na1—O5^ii^	88.13 (11)	Na1^vii^—O5—Na2	73.62 (10)
O6^i^—Na1—O3^i^	64.99 (11)	As2—O6—Mn1	146.46 (18)
O1^ii^—Na1—O3^i^	93.26 (11)	As2—O6—Na1^vi^	111.42 (15)
O2^iii^—Na1—O3^i^	91.88 (11)	Mn1—O6—Na1^vi^	101.49 (12)
O5^ii^—Na1—O3^i^	162.80 (13)	As2—O6—Na2	99.17 (13)
O6^i^—Na1—O7^iv^	85.65 (11)	Mn1—O6—Na2	95.37 (12)
O1^ii^—Na1—O7^iv^	159.09 (13)	Na1^vi^—O6—Na2	74.75 (11)
O2^iii^—Na1—O7^iv^	125.53 (12)	As1—O7—Mn1	111.73 (14)
O5^ii^—Na1—O7^iv^	66.66 (10)	As1—O7—Na2^vi^	165.60 (16)
O3^i^—Na1—O7^iv^	99.82 (11)	Mn1—O7—Na2^vi^	81.17 (10)
O3—Na2—O2^v^	85.11 (12)	As1—O7—Na1^x^	90.74 (12)
O3—Na2—O7^i^	104.26 (12)	Mn1—O7—Na1^x^	139.29 (14)
O2^v^—Na2—O7^i^	137.92 (14)	Na2^vi^—O7—Na1^x^	74.98 (10)
O3—Na2—O5	172.25 (14)	As2—O8—Mn1^i^	125.11 (15)
O2^v^—Na2—O5	87.16 (12)	As2—O8—Mn2^viii^	107.14 (14)
O7^i^—Na2—O5	81.95 (11)	Mn1^i^—O8—Mn2^viii^	125.73 (13)
O3—Na2—O1	79.48 (11)	As2—O8—Na2	93.68 (12)
O2^v^—Na2—O1	66.91 (11)	Mn1^i^—O8—Na2	74.88 (10)
O7^i^—Na2—O1	154.63 (13)	Mn2^viii^—O8—Na2	118.54 (13)
O5—Na2—O1	96.92 (11)		

Symmetry codes: (i) −*x*+1, *y*+1/2, −*z*+1/2; (ii) −*x*, *y*+1/2, −*z*+1/2; (iii) −*x*, −*y*+1, −*z*+1; (iv) *x*, *y*+1, *z*; (v) *x*, −*y*+1/2, *z*−1/2; (vi) −*x*+1, *y*−1/2, −*z*+1/2; (vii) −*x*, *y*−1/2, −*z*+1/2; (viii) *x*, −*y*+1/2, *z*+1/2; (ix) −*x*+1, −*y*+1, −*z*+1; (x) *x*, *y*−1, *z*.
